# Determinants of implanon discontinuation among women in Gedeo Zone, South Ethiopia: a case–control study

**DOI:** 10.1186/s40834-024-00263-1

**Published:** 2024-05-13

**Authors:** Tihtina Bezabih, Selamawit Dires Agidew, Selamawit Semagn Kebede, Moges Mareg Belay, Ever Siyoum, Eden Ashenafi, Mahlet Birhane

**Affiliations:** 1 Department of Reproductive Health, School of Public Health, College of Medical and Health Sciences, Dilla University, Dilla, Ethiopia; 2 Department of Nutrition, School of Public Health, College of Medical and Health Sciences, Dilla University, Dilla, Ethiopia

**Keywords:** Implanon, Discontinuation, Family planning, Gedeo Zone, Case–control study, South Ethiopia

## Abstract

**Background:**

The main public health problem that has a significant impact on the high fertility rate, unintended pregnancies, and induced abortion is Implanon cessation. In addition to Implanon being underutilized in Ethiopia, the rate of cessation is higher among women who started using it. Nevertheless, little is known about the factors that lead to Implanon cessation.

**Objectives:**

The study was aimed to determine the factors that led women to discontinue Implanon in Gedeo Zone, Southern Nation Nationalities and Peoples Region, southern Ethiopia, 2019.

**Method:**

A community-based unmatched case–control study design was carried out. Cases and controls were selected by a multi-stage sampling with a 1:2 case-to-control ratio. Data was entered into EPi-data version 3.1 and exported to Statistical Package for Social Sciences (SPSS) version 20 for analysis. A bivariate and multivariable logistic regression was used to identify the factors affecting Implanon discontinuation. The adjusted odd ratio (AOR) with 95% Confidence Interval (CI) was used to determine the precision of estimates, and statistical significance was declared at a *p*-value of 0.05.

**Result:**

From 516 women included in to the study, 495 (cases = 166 and controls = 329) women have responded to the questionnaires making a response rate of 96%. Monthly income 500—1000 birr [AOR: 0.3; 95% CI (0.2–0.5)], above 1000 birr [AOR: 0.2; 95% CI (0.1–0.4)], history of abortion [AOR: 2.0; 95% CI (1.0–4.1)], birth spacing [AOR: 0.6; 95% CI (0.3–0.9)], partner objection [AOR: 2.4; 95% CI (1.4–4.2)] were significantly associated factors.

**Conclusion:**

This study identified that monthly income, having abortion history, birth spacing, partner objection were the factors that affect Implanon discontinuation. To reduce implanon discontinuation, reducing abortion, involving partner and increasing the birth spacing, appropriate counseling before the insertion and appropriate follow-up by a health care practitioner are highly recommended.

## Background

Implanon is a three-year reversible single-rod progestin-only contraception that has a high level of contraceptive effectiveness [1]. Its ability to prevent pregnancy is accomplished by a number of mechanisms, including ovulation suppression, increased cervical mucus viscosity, and endometrial changes [2].

Implanon discontinuation is defined as discontinuation at before 3 years after insertion of Implanon [3]. It is becoming a major public health concern in different parts of the world and reaches up to 50% before three years of insertion even in developed countries [4, 5].

The use of Implanon has reportedly been discontinued due to a number of decisive variables.

These variables include, among others, poor care, a lack of follow-up, poor counseling by medical personnel, Implanon side effects, a desire for additional children, partner rejection, and more [6, 7]. The discontinuation of the long acting reversible contraceptives contributes to the high fertility rate, an unwanted pregnancy, and an induced abortion [8, 9]. Implanon is designed for 3 years of use to obtain the optimal benefits [8].

Implanon discontinuation is high in many regions of the world. For instance, a retrospective chart audit of 976 women who underwent implant surgery in Australia found that 50% of them stopped using Implanon after two years [5]. Approximately 67.3% of research participants had withdrawn their Implanon in the first year of use, while 94.4% had done so after the second year, according to another study carried out in Buffalo City Metropolitan Municipality, South Africa [6].

Ethiopia is one of the emerging nations with the worst discontinuation rates. Since 2009, counseling, Implanon insertion, and follow-up services have been offered at the level of the health post and household by Health Extension Workers (HEWs) who work for Ethiopia's federal ministry of health (FMoH). Despite this effort to transfer the task, Implanon utilization is poor and the discontinuation rate is significant [10, 11].

For instance, according to Ethiopian Demographic and Health Survey (EDHS) 2016 only 5.7% reproductive age women in Ethiopia used implants [12]. A study conducted in the Arsi zone revealed that about 80% of the study subjects removed their Implanon before 2.5 years of insertion. Similarly, in a study conducted by the Debre Markos town administration, the overall discontinuation rate in the last three years was found to be 46.5%. Early Implanon discontinuation rates were 65% and 23.4%, respectively, in other similar studies conducted in Debre Tabor town and Dale district in Southern Ethiopia [7, 13–15].

Moreover, this high rate of contraceptive discontinuation results in millions of women having an unmet need for contraception. In developing countries, about 225 million women are estimated to have an unmet need for modern methods of contraception and contribute to about 74 million unintended pregnancies, 290,000 maternal deaths, and 2.9 million newborn deaths every year. In another word, contraceptive discontinuation accounted for one-third (33%) of unintended recent births and 35% of unintended recent pregnancies in developing countries [16, 17].

Even though contraceptive implants are the most effective family planning methods available and well-accepted worldwide with proven long term protection, only 8% of married women in Ethiopia use implants and only 8% reproductive age women use Implants in Southern Nations, Nationalities, and peoples’ Regional State (SNNPRS) [12]. According to Gedeo Zone Health Department report of 2018, the long acting contraceptive coverage was 21% and 452 women have removed their implants in the first six months of insertion [18]. A part from its low utilization, Implanon discontinuation is common with high contribution to unplanned pregnancy, abortions, and maternal deaths [7]. Therefore identifying the determinants of Implanon discontinuation is important for health service programmers, planners and policy makers to improve the overall health of reproductive age women.

## Method

### Study design, and setting

A community based un–matched case–control study design was carried out from February 21st-April 21st/2019.The study was conducted at Gedeo Zone, Southern Nation, Nationality, Peoples region of Ethiopia. Gedeo Zone is one of the fourteen administrative zones in SNNPR of Ethiopia. Its capital town Dilla is found 365 km far away from Addis Ababa. It has six districts and two town administrations with a total population size of 1,166,163. The total number of reproductive age women are 271,7169 (23.3%) [19]. There are 146 health posts, 38 government health centers, 3 primary care hospitals and one referral hospital in the zone. Health posts provide only Implanon insertion service but, health centers, primary care hospitals, and referral hospital, provide both Implanon insertion and removal services.

### Population and participants

Cases-were all reproductive-age women (15–49 years) in Gedeo Zone who had discontinued their Implanon before 3 years of insertion and controls-were all reproductive-age women (15–49 years) who used Implanon for a completed 3 years.

### Sample size determination and sampling procedure

The sample size was determined assuming a 95% confidence level, an 80% power, the minimum detectable OR, and the proportion of controls exposed from different pieces of literature by using open-Epi version 3.1 with a case to controls ratio of 1: 2 as [7, 14, 15, 20]. The largest sample size was obtained when the follow-up appointment was assumed as a determinant. Considering a conventional design effect of 2 for multistage sampling and adding 10% of the potential nonresponses rate, the total sample size was 516 (172 for cases and 344 for controls). We have hierarchically selected the districts, the catchments and the study participants using a multistage sampling. First, out of the six districts and two town administrations of Gedeo Zone, two districts and one town administration were randomly selected (Bule district, Yirgacheffe district, and Dilla town). Then from the selected districts and town administration 7 health center catchments was selected using a simple random sampling (2 from Bule district, 4 from Yirgacheffe district, and 1from Dilla town).

The study participants (cases and controls) were selected from the sampling frame prepared from family planning registration book and long-acting family planning removal registration book of Health centers by simple random sampling. Women who were unable to give information and women with hysterectomy were excluded from the study.

### Study variables

The outcome of the study was Implanon discontinuation, and the Independent variables were Socio-economic and demographic characteristics (age, marital status, occupation, place of residence, educational status of women, educational status of husband/partner and income); obstetric factors(number of children, parity, and abortion); social factors (husband opposition, male involvement); Method related factors (side effects, intention to use, past contraceptive utilization, desire for pregnancy, the reason for choosing); family planning service-related factors (counseling, follow-up, perceived satisfaction, decision making, place of insertion, information about family planning, past contraceptive history).

### Data collection instrument

Data was collected using a semi-structured and interview administered questionnaire. It was prepared after reviewing different kinds of literature and has three parts. The questionnaire was prepared in English and translated to local language and back retranslated to English to check the consistency. Data collection was carried out by ten diploma nurses and was supervised by two degree nurses. Two-day training was given for data collectors and supervisors on the objective of the study, the contents of the questionnaire, and a timely collection of data. The data was collected from selected cases and controls through the house to house interview. In the absence of the women in her home, up to two attempts were made for interview and the respondent who were absent at the second visit were replaced from sampling frame randomly to lower the non-response rate. Study subjects who refused to participate in the study were considered as non-respondent. A pre-test of the questionnaire was carried out on 26 (5%) of the sample (9 cases and 17 controls) of population not included in the actual study population (Dilla zuria district) before the actual data collection period. The collected data was checked daily by the principal investigator, supervisors, and data collectors and investigators.

#### Operational definitions

##### Implanon discontinuation

Removal of Implanon by health professionals before 3 years of utilization.

##### Cases

Implanon user women who have discontinued Implanon utilization at a health facility before 3 years of insertion with the registered date of insertion and removal.

##### Controls

Implanon user women who have used Implanon for complete 3 years of insertion.

##### Side effect

When the women develop at least one side effect after Implanon insertion like menstrual disruption, insertion arm pain, headache, acne, and others.

##### Partner involvement

A husband accompanies women in the health facility during Implanon insertion.

##### Menstrual irregularity

Any deviation of a women’s regular menstrual cycle.

##### Prolonged menstrual bleeding

Menstrual flow lasting more than 7 consecutive days.

##### Heavy menstrual bleeding

Menstrual blood flow which client perceives as larger in amount than the usual.

##### Health Concerns

Previously existed physiological illness or medically known condition, which may result in fear of the use of Implanon contraceptive methods.

##### Misconception

Without scientific evidence, the women perceived Implanon contraceptive method can cause infertility, paralysis and shifting to other sites etc.

##### Birth spacing intention

Women who are using contraceptive to wait two or more years before having their next child.

##### Birth limiting intention

Women who are using contraceptive to avoid the birth of any more children.

### Data quality control

To assure the quality of data, a pre-test of the questionnaire was carried out on 26 (5%) of the sample (9 cases and 17 controls) of population not included in the actual study population (Dilla zuria district) before the actual data collection period. Based on the pretest, a questionnaire was corrected to ensure clarity, wording, logic sequence, and skip patterns. The final version of the questionnaire was translated into the local language of the respondents (Gedeufa language) and was used for the data collection. Training on how to administer the data collection process was given for all data collectors and supervisors before the process of data collection for two days. Data completeness was checked every day.

### Data processing, analysis

First, the data were checked for their completeness and consistency and entered into Open Epi version 3.1exported to SPSS statistical software version 20 for analysis. Descriptive statistics were used to measure the characteristics of the study participants. Both bivariate and multivariable logistic regression analysis were computed to identify factors associated with implanon discontinuation. Variables with a P-value of less than 0.25 in a bivariate analysis were candidates for multivariable logistic regression. In the multivariable analysis, a variable with a *P* value of ≤ 0.05 was considered as having statistically significant association with implanon discontinuation. The level of the association was measured by using odds ratio (OR) and corresponding 95% confidence interval (CI). The result was presented using text, chart, and figures.

## Result

### Socio-demographic characteristics

About 495 women had responded to the questionnaires with a response rate of 96.5% among cases and 95.6% among controls. The mean (± SD) age of the study participants was 29.1 (± 6.1) years: 29.1 (± 6.4) for cases and 29.2 (± 5.5) years for controls with age ranging from 16 to 44 years (16 to 40 years for cases and 18 to 44 years for controls). Most of cases 56 (33.7%) as well as controls 88 (26.7%) were in 30–34 years category.

Majority of cases (89.8%) and controls (92.4%) were married. While, 6.0% of cases and 4.0% of controls were single, and 4.2% of cases and 3.6% of controls were divorced and widowed. Large proportion of cases (72.9%) and controls (64.7%) had no formal education, 16.3% of cases and 20.7% of controls had primary education, whereas 26.5% of cases and 14.6% of controls had secondary and above education. More than half of husbands of the cases (54.2%) and less than half of the controls (46.8%) had no formal education. Large proportions of cases (43.4%) as well as controls (42.2%) were housewives.

The mean (± SD) monthly income of the study participants was 742.5 (± 518.9) Ethiopian Birr (ETB): 612.9 (± 474.6) for cases and 807.9(± 528.6) ETB for controls. More than half (63.3%) of cases and about 40.1% controls earn below 500 ETB. The majority of cases (84.3%) and controls (84.2%) were from a rural area but the remaining 15.7% of cases and 15.8% of controls were from the urban area (Table 1).

**Table 1:** Socio-demographic characteristics of women who used Implanon in Gedeo Zone, Southern Nation, nationalities, and peoples region of Ethiopia, 2019 (*n* = 495)

Variables		Cases (*n* = 166) n (%)	Controls (*n* = 329) n (%)	Total (*n* = 495) n (%)
Age in years	Mean(± SD)	29.1(± 6.4)	29.2(± 5.5)	29.1(± 6.1)
	15–19	13(7.8)	33(10.0)	46(9.3)
	20–24	17(10.2)	58(17.6)	75(15.2)
	25–29	50(30.1)	74(22.5)	124(25.1)
	30–34	56(33.7)	88(26.7)	144(29.1)
	≥ 35	30(18.1)	76(23.1)	106(21.4)
Religion	Protestant	112(67.5)	268(81.5)	380(76.8)
	Orthodox	32(19.3)	38(11.6)	70(14.1)
	Muslim	12(7.2)	19(5.8)	3 (6.3)
	Catholic	10(6.0)	4(1.2)	14(2.8)
Ethnicity	Gedeo	107(64.5)	252 (76.6)	359(72.5)
	Oromo	24(14.5)	31(9.4)	55(11.1)
	Amhara	13(7.8)	21(6.4)	34(6.9)
	Gurage	17(10.2)	16(4.9)	33(6.7)
	Others^a^	5(3.0)	9(2.7)	14(2.8)
Marital status	Single	10(6.0)	13(4.0)	23(4.6)
	Married	149(89.8)	304(92.4)	453(91.5)
	Others^b^	7 (4.2)	12(3.6)	19(3.8)
Educational status of women	No formal education	121(72.9)	213(64.7)	334(67.5)
	primary	27(16.3)	68(20.7)	95(19.2)
	Secondary and above	18(10.8)	48(14.6)	66(13.3)
Educational status of husband/partner	No formal education	90(54.2)	154(46.8)	244(49.3)
	primary	32(19.3)	86(26.1)	118(23.8)
	Secondary and above	44(26.5)	89(27.1)	133(26.9)
Occupational status of women	Housewife	72(43.4)	139(42.2)	211(42.6)
	Farmer	35(21.1)	101(30.7)	136(27.5)
	Merchant	37(22.3)	51(15.5)	88(17.8)
	Government employer	11(6.6)	24(7.3)	35(7.1)
	Other ^c^	11(6.6)	14(4.3)	25(5.1)
Monthly income	Mean(± SD)	612.9 (± 474.6)	807.9(± 528.6)	742.5 (± 518.9)
	< 500	105(63.3)	132(40.1)	237(47.9)
	500–1000	43(25.9)	135(41.0)	178(36.0)
	> 1000	18(10.8)	62(18.8)	60(16.2)
Residence	Urban	26(15.7)	52(15.8)	78(15.8)
	Rural	140(84.3)	277(84.2)	417(84.2)

^a^Kembta, silte and Wolayita

^b^Divorced and widowed

^c^Daily laborer, private employer and student; and n = number

### Obstetric characteristics

One hundred fifty-seven (94.6%) cases and 316(96%) controls had given birth while 9(5.4%) cases and 13(4.0%) controls were nulliparous. Out of those who had given birth 115 cases (69.3%) and 219 controls (66.6%) had one to four alive children. While, 42 cases (25.3%) and 97 controls (29.4%) had more than five alive children. Among the respondents, 31cases (19.7%) and 23 controls (7.3%) had abortion history.

### Family planning service-related characteristic

About 128 (77.1%) cases and 263 (79.9%) controls had heard contraception before Implanon insertion. Majority of the participant 108 (65.1%) cases and 236 (71.7%) controls ever used modern contraceptive before Implanon. Out of these women 83 (76.9%) cases and 160 (67.9%) controls ever used injectable followed by oral contraceptive pills in 23 (21.3%) cases and 71(30.0%) controls. Almost three-quarters of participants 122 (73.5%) cases and 258 (78.4%) controls had ever heard Implanon before insertion. Major source of information in 72 (59.0%) cases and 157(60.9%) controls were health extension workers followed by health care workers in 27(22.1%) cases and 65 (25.2%) controls.

About 118 cases (71.1%) and 211 controls (64.1%) got counseling service during Implanon provision. Among those who got counseling service, 16 (13.6%), 57 (48.3%), 45 (38.1%), 51(43.2%) cases and 79 (37.4%), 161(76.3%), 162 (76.8%), 149 (70.6%) controls were counseled on the effectiveness, duration of action, advantage, and side effect of the method, respectively. Among those who got counseling on possible side effects, majority of cases (73.1%) and controls (81.8%) were counseled on menstrual irregularity.

Ninety-two cases (55.4%) and 225 controls (68.4%) have had a follow-up. About 74 cases (44.6%) and 104 controls (31.6%) did not have follow-up visits during their Implanon utilization period. More than half (57.8%) cases and (45.6%) controls had not satisfied by the service given. Majority of cases (73.5%) and controls (84.2%) intention of Implanon use was birth spacing. More than half of the cases (53.0%) and controls (51.1%) took the method from health post and health center, respectively.

Majority of the cases (60.8%) and controls (47.4%) had decided to use the method by themselves. About 25.3% of cases and 11.9% of controls had faced objection from their husband/partner. A large proportion of cases (88.0%), as well as controls (83.3%), had not accompanied by their husband/partner during Implanon insertion (Table 2).

**Table 2:** Family planning service related characteristic of women who used Implanon in Gedeo Zone, Southern Nation, nationalities, and peoples region of Ethiopia from February 21st-April 21st, 2019 (*n* = 495)

**Variables**		**Cases (*n* = 166) (%)**	**Controls (*n* = 329) (%)**	**Total (*n* = 495) (%)**
Information on any contraception before Implanon insertion	Yes	128 (77.1)	263 (79.9)	391(79.0)
	No	38 (22.9)	66 (20.1)	104 (21.0)
Ever used modern contraceptives before Implanon	Yes	108 (65.1)	236 (71.7)	344 (69.5)
	No	58 (34.9)	93 (28.3)	151(30.5)
Ever heard about Implanon before used	Yes	122 (73.5)	258 (78.4)	380 (76.8)
	No	44 (26.5)	71(21.6)	115 (23.2)
Source of information (*n* = 380)	Health extension workers (HP)	72(59.0)	157(60.9)	229 (60.3)
	Health workers (HC)	27 (22.1)	65 (25.2)	92 (24.2)
	Friends	21(17.2)	31(12.0)	52 (13.7)
	Media	2 (1.6)	5 (1.9)	7 (1.8)
Pre-insertion counseling	yes	118 (71.1)	211(64.1)	329 (66.5)
	No	48 (28.9)	118(35.9)	166 (33.5)
Information obtained during counseling *n* > 495	Effectiveness	16 (13.6)	79(37.4)	95 (28.9)
	Duration of action	57(48.3)	161(76.3)	218 (66.3)
	Advantage	45(38.1)	162 (76.8)	207 (62.9)
	Side effect	51(43.2)	149 (70.6)	200 (60.8)
Possible side effects counseled (*n* = 200)	Menstrual irregularity	38 (73.1)	121(81.8)	159 (79.5)
	Weight change	3 (5.8)	15 (10.1)	18 (9.0)
	Headache	5 (9.6)	8 (5.4)	13 (6.5)
	Other ^e^	6 (11.5)	4 (2.7)	10 (5.0)
Follow up	Yes	92 (55.4)	225 (68.4)	317 (64.0)
	No	74 (44.6)	104 (31.6)	178 (36.0)
Perceived satisfaction	Yes	70 (42.2)	179 (54.4)	249 (50.3)
	No	96 (57.8)	150 (45.6)	246 (49.7)
Intention of Implanon use	Spacing	122 (73.5)	277 (84.2)	399 (80.6)
	Limiting	35 (21.1)	43 (13.1)	78 (15.8)
	Delaying	9 (5.4)	9 (2.7)	18 (3.6)
Place of insertion	Hospital	6 (3.6)	7 (2.1)	13 (2.6)
	Health center	72 (43.4)	168 (51.1)	240 (48.5)
	Health post	88 (53.0)	154 (46.8)	242 (48.9)
Main decider	My self	101(60.8)	156 (47.4)	257 (51.9)
	with husband/partner	42 (25.3)	125 (38.0)	167 (33.7)
	Other ^f^	23 (13.9)	48 (14.6)	71(14.3)
Partner objection	No	124 (74.7)	290 (88.1)	414(83.6)
	Yes	42 (25.3)	39 (11.9)	81(16.4)
Partner accompanied during Implanon insertion	Yes	20 (12.0)	55 (16.7)	75 (15.2)
	No	146 (88.0)	274 (83.3)	420 (84.8)
Faced side effect	No	65 (39.2)	154 (46.8)	219 (44.2)
	Yes	101(60.8)	175 (53.2)	276 (55.8)
Side effect type faced (*n* = 276)	Menstrual irregularity	63 (62.4)	99 (56.6)	162 (58.7)
	Weight gain	29 (28.7)	45 (25.7)	74 (26.8)
	Other ^g^	9 (8.9)	31(17.7)	40 (14.5)
Menstrual irregularity type faced (*n* = 162)	Amenorrhea	4 (6.3)	30 (30.3)	34 (21.0)
	Prolonged bleeding	12 (19.0)	30 (30.3)	42 (25.9)
	Heavy bleeding	31(49.2)	23 (23.2)	54 (33.3)
	Frequent spotting and inter menstrual bleeding	16 (25.5)	16 (16.2)	32 (19.8)

^d^Condom and IUCD

^e^Arm pain and mood change

^f^Husband and health provider

^g^Arm pain, headache and mood change

### Reason for discontinuation

From the total of 166 cases, the majority (50.0%) had discontinued Implanon due to side effect, (17.5%) due to desire for more children, and (7.8%) of them discontinued due shifted to other family planning methods (Table 3).

**Table 3:** Reason for Implanon discontinuation of women who used Implanon in Gedeo zone Southern Nation, nationalities, and peoples region of Ethiopia from February 21st-April 21st, 2019 (*n* = 495)

Reason		Frequency (*n* = 166 (%))
Due to side effect		83 (50.0)
Health concerns		17 (10.2)
Husband opposition		17 (10.2)
Misconception		7 (4.2)
Side effect type for discontinuation (*n* = 83)	Menstrual irregularity	57 (68.7)
	Weight gain	24 (28.9)
	Others ^h^	2 (2.4)
Menstrual irregularity type for discontinuation (*n* = 57)	Heavy bleeding	28 (49.1)
	Frequent spotting	14 (24.6)
	Prolonged bleeding	10 (17.5)
	Amenorrhea	4 (7.0)
	Inter menstrual bleeding	1 (1.8)

^h^headache and arm pain

### Determinants of implanon discontinuation

In the multivariable logistic analysis monthly income, history of abortion, Ever heard about Implanon before used, pre-insertion counseling, information on effectiveness¸ duration of action, advantage and side effect, follow up, the intention of Implanon use, partner objection and menstrual irregularity type faced had significantly associated with Implanon discontinuation.

Women who earned a monthly income of 500–1000 ETB and over 1000 ETB had 70% and 80% less likely to discontinue Implanon [AOR: 0.3; 95% CI (0.2–0.5)] and AOR: 0.2; 95% CI: (0.1–0.4)], respectively as compared to women who earned below 500 ETB. The odds of Implanon discontinuation were 2.0 times greater among women who had a history of abortion as compared to women who did not experience abortion [AOR: 2.0; 95% CI (1.02–4.1)].

The women who never heard about Implanon before insertion had 2.4 times more likely discontinue Implanon as compared to those who ever heard about Implanon before insertion [AOR: 2.4; 95% CI (1.3–4.6)]. Similarly women who without pre-insertion counseling had 10.9 times more likely discontinue Implanon as compared to those who had got counseling service [AOR: 10.9; 95% CI (3.4–34.9)]. The odds of Implanon discontinuation were 7.8, 4.1, 5.2 and 4.7 times higher among women without counseling on effectiveness¸ duration of action, advantage and side effect, respectively as compared to those who had counseling on respective determinants. Women counseled on menstrual irregularity had 60% reduced odds Implanon discontinuation [AOR: 0.4; 95% CI (0.2–0.9)] as compared to women who had counseled on other side effects like weight gain, headache, and mood change.

The odds of Implanon discontinuation among women who were not scheduled for follow-up visits were 70% higher than those who were appointed for follow-up [AOR: 1.7; 95% CI (1.1–2.7)]. Additionally, women who had birth spacing intention were 40% less likely to discontinue Implanon [AOR: 0.6; 95% CI (0.3–0.9)] as compared to women who had birth limiting intention. Women who had faced partner objection had 2.4 times increased odds of Implanon discontinuation [AOR: 2.4; 95% CI (1.4–4.2)] as compared to those who had not faced objection. Women who had faced heavy bleeding had 3.2 times increased odds of Implanon discontinuation [AOR: 3.2; 95% CI (1.4–7.2)] as compared to women who had faced other menstrual irregularities (amenorrhea and frequent spotting) (Table 4).

**Table 4:** Determinants of Implanon discontinuation among women who used Implanon in Gedeo zone Southern Nation, nationalities, and peoples region of Ethiopia, 2019 (*n* = 495)

Variables		Cases *n*(%)	Controls *n*(%)	COR (95%CI)	AOR(95%CI)
Age in years	15–19	13(7.8)	33(10.0)	1.0(0.5–2.2)	0.6(0.2–1.7)
	20–24	17(10.2)	58(17.6)	0.7(0.4–1.5)	0.7(0.3–1.7)
	25–29	50(30.1)	74(22.5)	1.7(0.9–3.0)	1.1(0.5–2.4)
	30–34	56(33.7)	88(26.7)	1.6(0.9–2.8)	0.9(0.47–1.9)
	≥ 35	30(18.1)	76(23.1)	1	1
Educational status of women	No formal education	121(72.9)	213(64.7)	1.5(0.8–2.7)	0.9(0.4–2.3)
	Primary	27(16.3)	68(20.7)	1.1(0.5–2.1)	0.5(0.2–1.3)
	Secondary and above	18(10.8)	48(14.6)	1	1
Monthly income	< 500	105(63.3)	132(40.1)	1	1
	500–1000	43(25.9)	135(41.0)	0.4(0.3–0.6) ^∗^	0.3(0.2–0.5) ^∗∗^
	> 1000	18(10.8)	62(18.8)	0.4(0.2–0.7) ^∗^	0.2(0.1–0.4) ^∗∗^
History of abortion	No	126(80.3)	293(92.7)	1	1
	Yes	31(19.7)	23(7.3)	3.1(1.8–5.6) ^∗^	2.0(1.0–4.1) ^∗^
Ever heard about Implanon before used	Yes	122(73.5)	258(78.4)	1	1
	No	44(26.5)	71(21.6)	1.3(0.9–2.0)	2.4(1.3–4.6) ^∗^
Pre-insertion counseling	Yes	118(71.1)	211(64.1)	1	1
	No	48(28.9)	118(35.9)	0.7(0.5–1.1)	10.9(3.4–34.9) ^∗∗^
Type of counseling	Individual	79(66.9)	128(60.7)	1.7(0.9–3.0)	0.7(0.3–1.6)
	Mass	19(16.1)	28(13.3)	1.9(0.9–4.1)	0.7(0.3–1.9)
	With husband	20 (16.9)	55(26.1)	1	1
Counseling on effectiveness	Yes	16(13.6)	79(37.4)	1	1
	No	102(86.4)	132(62.6)	3.8(2.1–6.9) ^∗^	7.8(3.4–17.7) ^∗∗^
Counseling on duration of action	Yes	57(48.3)	161(76.3)	1	1
	No	61(51.7)	50(23.7)	3.5(2.1–5.6) ^∗^	4.1(2.2–7.7) ^∗∗^
Counseling on advantage	Yes	45(38.1)	162(76.8)	1	1
	No	73(61.9)	49(23.2)	5.4(3.3–8.8) ^∗^	5.2(2.9–9.4) ^∗∗^
Counseling on side effect	Yes	51(43.2)	149(70.6)	1	1
	No	67(56.8)	62(29.4)	3.2(1.9–5.1) ^∗^	4.7(2.4–9.1) ^∗∗^
Possible side effects counseled	Menstrual irregularity	38(73.1)	121(81.8)	0.6(0.3–1.3)	0.4(0.2–0.9) ^∗^
	Others	14(26.9)	27(18.2)	1	1
Follow up	Yes	92(55.4)	225(68.4)	1	1
	No	74(44.6)	104(31.6)	1.7(1.2–2.6) ^∗^	1.7(1.1–2.7) ^∗^
Perceived satisfaction	Yes	70(42.2)	179(54.4)	1	1
	No	96(57.8)	150(45.6)	1.6(1.1–2.4) ^∗^	1.2(0.8–1.9)
Intention of Implanon use	Spacing	122(73.5)	277(84.2)	0.5(0.3–0.9) ^∗^	0.6(0.3–0.9) ^∗^
	Limiting	35(21.1)	43(13.1)	1	1
	Delaying	9(5.4)	9(2.7)	1.2(0.4–3.4)	1.6(0.5–4.8)
Main decider	My self	101(60.8)	156(47.4)	1.9(1.3–2.9) ^∗^	1.5(0.9–2.5)
	with husband/partner	42(25.3)	125(38.0)	1	1
	Other	23(13.9)	48(14.6)	1.4(0.8–2.6)	1.1(0.6–2.1)
Partner objection	No	124(74.7)	290(88.1)	1	1
	Yes	42(25.3)	39(11.9)	2.5(1.6–4.1) ^∗^	2.38(1.6–4.2) ^∗^
Partner accompanied during Implanon insertion	Yes	20(12.0)	55(16.7)	1	1
	No	146(88.0)	274(83.3)	1.5(0.9–2.5)	0.9(0.5–1.8)
Faced side effect	No	65(39.2)	154(46.8)	1	1
	Yes	101(60.8)	175(53.2)	1.4(0.9–2.0)	0.8(0.3–1.9)
Side effect type faced	Menstrual irregularity	63(62.4)	99(56.6)	2.2(0.9–4.9)	2.4(0.9–6.5)
	Weight gain	29(28.7)	45(25.7)	2.2(0.9–5.3)	2.3(0.9–5.8)
	Other	9(8.9)	31(17.7)	1	1
Menstrual irregularity type faced	Other	20(31.7)	46(46.5)	1	1
	Prolonged bleeding	12(19.0)	30(30.3)	0.9(0.4–2.2)	0.9(0.4–2.3)
	Heavy bleeding	31(49.2)	23(23.2)	3.1(1.5–6.6) ^∗^	3.2(1.4–7.2) ^∗^

*Statistically significant at *p* < 0.05 and **Statistically significant at *p* < 0.001

## Discussion

Most effective and long-acting contraceptive like Implanon has been confirmed as a good fertility control measure to prevent unintended pregnancy with a view to reducing high fertility and maternal mortality, and improve child health. However, this becomes unrealistic where women who are at risk of pregnancy do not use or shortly discontinued it due to different determinant factors. This study found that as the monthly income of the women increases, the likelihood of Implanon discontinuation decreases. This finding is consistent with the study done in Dale district, south Ethiopia [15]. The possible explanation for this finding might be that women who had relatively higher income are better educated than those with less income. Education is helpful to understand and outweigh the advantage and disadvantage of the methods they are using hence; good continuation of Implanon might be observed [3, 21].

History of abortion was significantly associated with Implanon discontinuation in the study. This result was consistent with a study in Diguna Fango district, Wolayita Zone, South Ethiopia of which Implanon discontinuation was 2.3 times higher among women who had a history of previous abortion as compared to women who have not history of abortion. Women who had a history of abortion might have fear of childlessness in the double burden of abortion and contraception [3].

This study indicated that Implanon discontinuation was more common among women who never heard about Implanon before insertion, who without pre-insertion counseling, and counseling on (effectiveness¸ duration of action, advantage, and side effect). Women who were counseled on menstrual irregularity had 60% less likely to have Implanon discontinuation in comparison to women who were counseled on other side effects like weight gain, headache, and mood change. This finding is consistent with the study done in Dale district, South Ethiopia [15], Diguna Fango district, Wolayita Zone [3], Debre Tabor [7], Debre Markos [14], and Mekelle town [21]. However, a different study done in Ofla district, Tigray, Northern Ethiopia [20], reported that counseling about benefit and effectiveness had no statistically significant association with Implanon discontinuation. This might be due to the fact that women who were informed on the effectiveness, advantage, and possible side effects, as well as women who made an informed choice of the method will tolerate minor changes (side effects), whereas those who were not informed will seek to remove it before 3 years.

This study showed that the odds of Implanon discontinuation among women who did not have a follow-up appointment were 70% higher than those who had an appointment for follow-up. If there is no follow-up the women might not get further counseling on side effects and support from health professionals. In addition, clients with a complaint of the side effect, especially, those who suffer from menstrual irregularities could not get supportive treatment from health care providers and hence they might not be encouraged to continue their Implanon use. This finding is also similar to studies done in Tigray [20], Debre Tabor [7], Dale [15], and Diguna Fango district [3]. While in Mekelle town study follow-up appointment had no statistically significant association [21].

This study also suggested that women who had birth spacing intention were 40% less likely discontinue Implanon as compared to women who had birth limiting intention. This finding is different from a study done in Jimma town which revealed that woman who wanted pregnancy spacing were 1.37 more likely discontinue their contraceptive use than women who wanted to limit pregnancy [22]. The difference was might be due to the age differences of the women included in these two studies. In Jimma town study, the majority of women (59.1%) were below 30 years while in this study about 50.5% of women above 30 years old. The other reason might be due to inadequate pre-insertion counseling particularly about the expected side effects which could result in discontinuation of Implanon while still in need.

Women who had faced partner objection had 2.4 times more likely to have Implanon discontinuation as compared to their counter parts. The possible explanation for this might be menstrual irregularities which can interfere with their social, cultural and sexual activities [23]. Wanting more children and lack of pre insertion counseling which includes their husband might also be another reason [24]. Women who had faced heavy bleeding had 3.16 times more likely to have Implanon discontinuation than their counterparts. The possible explanation for this might also be the interference with their daily activities and sexual relationships [20]. The finding of this study can be generalized to all Gedeo zone Implanon discontinuers since study participants were selected randomly. As a limitation there might recall bias, being a case control study; it can only tell us the presence of association but not causal relationship.

## Conclusion and recommendations

This study identified that determinants of Implanon discontinuation were monthly income, having history of abortion, awareness, pre-insertion counseling, counseling on (effectiveness¸ duration of action, advantage and side effect), having follow-up, intention of Implanon use, partner objection, and menstrual irregularity type faced (heavy bleeding). Pre insertion counseling with partners, continuous monitoring and early management of the side effects is recommended to reduce discontinuation of implanon. To reduce implanon discontinuation, reducing abortion, involving partner and increasing the birth spacing, appropriate counseling before the insertion and appropriate follow-up by a health care practitioner are highly recommended.

## Data Availability

All the data included in the manuscript can be accessed from the corresponding author upon request via memymine0@gmail.com.

## Abbreviations

CSA: Central Statistical Agency
DURH: Dilla University Referral Hospital
EDHS: Ethiopian Demographic and Health Survey
FMOH: Federal Ministry of Health
HEW: Health Extension Worker
IRB: Institutional Review Board
IUCD: Intra Uterine Contraceptive Device
PPS: Probability Proportional to Size
SRS: Simple Random Sampling
SSA: Sub- Saharan Africa
SNNP: Southern Nations Nationalities and Peoples
SPSS: Statistical Package for Social Sciences
